# Clinical Trial Accrual: Obstacles and Opportunities

**DOI:** 10.3389/fonc.2016.00103

**Published:** 2016-04-25

**Authors:** B. J. Rimel

**Affiliations:** ^1^Cedars-Sinai Medical Center, Los Angeles, CA, USA

**Keywords:** clinical trial accrual, gynecologic oncology, accrual, participation, enrollment

Less than 2% of patients diagnosed with cancer participate in a clinical trial in the United States (1). Gynecologic oncology patients do not appear to participate in trials with any more frequently than other cancer types. While gains in progression free survival have continued to improve overall life span for women with advanced gynecologic cancers, more cures have not been realized. Clinical trials, defined by the National Institute of Health as “a research study in which one or more human subjects are prospectively assigned to one or more interventions to evaluate the effects of those interventions on health-related biomedical or behavioral outcomes” (2), are the primary focus for enrollment of most patients with an active malignancy. Clinical research studies such as tissue banks and longitudinal cohort studies provide invaluable data for researchers but require a consenting population and may be overlooked when the focus is on therapeutic intent. Lack of accrual to clinical trials leads to early closure of studies and a waste of critical resources as well as extended periods of enrollment, which can hinder the ability to interpret the results. Stensland et al. reported that 1 in 4 cancer clinical trials were stopped early with 1 in 10 being stopped for poor accrual (3). A panel of experts convened by the NCI and ASCO to discuss barriers to clinical trial enrollment in 2013 (4) cited barriers in three areas as most significant: (1) patient/community, (2) physician/provider level, and (3) site/organizational. Physician/provider level barriers include willingness to refer a patient for study, lack of knowledge about available clinical trials, and concern regarding a patient’s ability to participate (4–6). Patient/community barriers have been noted to include being unaware of trial opportunities and complexity and stringency of the protocol (7).

But really, in this time of internet and social media, of immediate and total access to seemingly endless information, *why* are adult patients not enrolling on clinical trials? A large single institution review of clinical trial enrollment noted a dramatic difference in the proportion of pediatric cancer patients enrolled in clinical trials compared to adult (22 vs. 6%) (8). Is this because parents of children with cancer and young adults with cancer are so much better at searching the internet for clinical trial opportunities? Unlikely, this high rate of participation in the pediatric population preceded the internet age. I believe there are two vital differences between the adult and pediatric cancer communities. First, centralization of treatment in pediatric cancer results in high volume centers. The rarity of pediatric cancer forced pediatric oncologists to band together in universities and research centers. The vast majority of children and young adults are treated in these centers today. Data from several areas suggest that treatment in high volume centers results in improved pediatric oncology outcomes (8, 9). Still, most pediatric cancers are exceptionally rare. Even at high volume centers, collaboration with other sites must be done to gather enough similar cases for research.

Second, and perhaps most importantly, there is a pervasive culture in pediatric oncology that “clinical trials are standard practice in cancer treatment for children, adolescents, and young adults” (https://www.childrensoncologygroup.org/index.php/what-is-a-clinical-trial). The pediatric oncology community has remained faithful to the charge that cure is the goal (10). In addition, the research structure embraces that the cancers in this space are inherently rare and as such designed studies that use the available patient volume. These elements seem to combine to provide a complete package of physician motivation, patient engagement, and available studies for the vast majority of patients despite the low numbers.

In gynecologic oncology, there are no data to suggest that female gynecologic cancer patients participate in clinical trials at a higher rate. However, a recent large retrospective study by Cliby et al. suggests that treatment for ovarian cancer at high volume centers improves outcomes (11). The authors of this study suggest that a national effort be made to provide access to women to centers with expertise in ovarian cancer. It remains to be seen if these data will move patients from community settings into centers where participation in clinical trials is more common.

The National Comprehensive Cancer Network states on their website that “without clinical trials, cancer care can’t improve.” This group compiles clinical practice guidelines from the data produced by clinical trials to help guide the care for patients treated off study. Access to this resource is simple and available to both patients and providers. It is updated regularly and carefully curated.

This is in sharp contrast to information about open clinical trials. The main resource for clinical trials information is the national registry of clinical trials, which is available online at www.clinicaltrials.gov. The goal of the registry was to require the registration of all clinical trials in the US and to provide a resource for clinicians and patients to find open studies. The site is searchable by location and disease but is often woefully out of date. Studies that have closed months ago are often still listed as actively recruiting, studies that have published data are not listed as published, and studies that are open will often have incorrect listings of site information. This lack of timely and correct information results in the inability for interested patients and clinicians to find open and appropriate trials.

The barriers for women with cancer to participate in clinical trials are numerous but they are not insurmountable. Dramatic and sweeping cultural change is necessary to bring about rates of adult enrollment that rival the pediatric population. A profound commitment to the provision of timely data to the national clinical trials registry by sponsors and the timely curating of the website is required for better clinical trial information. A commitment to providing access to high volume centers with experience in gynecologic cancer is required for improving outcomes with the current available strategies. Lastly, physicians and patients need to fully commit themselves to a belief that clinical trial participation can really bring about better treatments and better drugs and most importantly, better lives.

## Author Contributions

The author confirms being the sole contributor of this work and approved it for publication.

## Conflict of Interest Statement

The author declares that the research was conducted in the absence of any commercial or financial relationships that could be construed as a potential conflict of interest.
